# Simulation‐based education for medical radiation students: A scoping review

**DOI:** 10.1002/jmrs.572

**Published:** 2022-02-17

**Authors:** Minh Chau, Elio Arruzza, Nathan Johnson

**Affiliations:** ^1^ UniSA Allied Health and Human Performance University of South Australia Adelaide South Australia Australia; ^2^ South Australia Medical Imaging Flinders Medical Centre Bedford Park South Australia Australia

**Keywords:** Education, medical radiation, radiography, simulation

## Abstract

Simulation‐based education is a significant aspect of teaching clinical skills in tertiary medical radiation science programmes, allowing students to experience the clinical setting in a safe environment. As an educational tool, simulation exists in many valid forms including role play, interprofessional simulation and virtual reality simulation. This scoping review looks at the current literature in this field to identify the evidence surrounding simulation‐based education for medical radiation students. The purpose of this review is to provide an evidence‐based guide for educators, identify gaps in the literature and suggest areas of future research. Data extraction was performed on 33 articles where the interventions could be categorised into either role play simulation, virtual simulation, simulation videos or online learning environments. Most studies demonstrated that simulation could improve clinical competence and increase preparedness and confidence for clinical placement. Student satisfaction remained high throughout the studies; however, it is the view of many that although simulation‐based education is a valid and effective tool, it is complementary to and not a replacement for clinical placement.

## Graphical abstract

This scoping review investigated the current literature in this field to identify the evidence surrounding simulation‐based education for medical radiation students. Student satisfaction remained high throughout the studies; however, it is the view of many that although simulation‐based education is a valid and effective tool, it is complementary to and not a replacement for clinical placement.

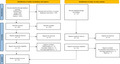

## Introduction

Clinical education is a core component of medical radiation university programmes (Medical Imaging/Diagnostic Radiography, Radiation Therapy and Nuclear Medicine) with simulation recognised as an essential preparatory tool for work‐integrated learning and clinical practice. Over the course of their undergraduate studies, students are required to develop a solid grounding in academic knowledge together with the associated technical and patient‐centred capabilities to facilitate a holistic approach in their own discipline. Globally, there is increasing pressure for training institutions to develop the competency of their students without the negative impacts that may be associated with clinical placements. This has resulted in university educators reassessing how to best facilitate the development of practical clinical skills in effective, safe and supported learning environments. Students not only need to be academically prepared for placement, but also need opportunities to develop technical skills outside the clinical learning environment.

Simulation‐based education is a highly effective tool for mimicking the clinical environment to teach skills to students and practitioners in healthcare.^1^ Founded on educational theories, a simulation program can provide training and professional development as well as opportunities for student assessment.^2^ All phases of the simulation, from preparation, pre‐briefing, the simulation activity, feedback, debriefing, to evaluation and reflection, play significant roles in the individuals’ learning.^3^ Of particular importance is the reflection process, with Levett‐Jones and Lapkin^4^ suggesting that the advantages of the debrief phase outweigh the actual simulation activity.

While virtual simulation has been successfully embedded within radiation therapy programs in Australia, the use of virtual simulation within diagnostic radiography has not been widely adopted despite some promising recent studies.^5^, ^6^ An Australian study confirmed the effectiveness of simulating clinical practice using anthropomorphic phantoms to develop patient positioning and communication skills.^7^ Another Australian study, Gunn and colleagues,^8^ demonstrated that virtual reality simulation is more effective at improving clinical skills than conventional teaching methods. In addition, other studies have shown that medical radiation students benefit from simulation in an interprofessional context, resulting in improved confidence, teamwork and preparedness.^9^, ^10^, ^11^ A systematic review concluded that simulation training increased students’ knowledge, confidence and satisfaction.^12^ Students value simulation training because they can see, practise and perform techniques/skills that may not be possible while on placement.

Despite the recent studies conducted in this field, many educators continue to use conventional teaching methods rather than seeking the potential benefits that simulation has to offer. Student preparation for clinical practice is essential and should be conducted with the most appropriate teaching methods to achieve the best results. Several scoping reviews and meta‐analyses have been performed in the field of nursing and medicine. There is, however, a scarcity of comprehensive literature review on this contemporary pedagogical approach. It is also unknown if medical radiation simulation curricula have been designed according to current best practice guidelines incorporating the cycle of simulation phases. The aim of this scoping review is to provide a contemporary evidenced‐based guide to simulation‐based education in medical radiation programs.

## Materials and Methods

A scoping review was performed to assess the current literature on the use of simulation for medical radiation students in an academic setting. Our existing knowledgebase and initial literature review of this topic have discovered a wide variety of alternate approaches to simulation education in medical radiation science. These aspects differ particularly in terms of the setting, duration and technology utilised by educators. Scoping reviews are particularly useful in this case, especially as our topic exhibits a complex and heterogeneous nature not amenable to a more precise form of review.^14^ Overall, this review was intended to ‘map out’ the current literature, attempting to explore the conceptual boundaries of the topic and provide a clear indication of the volume of literature and an overview of its focus.

The organisational framework described by Arksey and O'Malley^13^ was chosen as the preferred method in evaluating the extent of available evidence for this mapping overview. Specifically, this method entails: (1) identifying the research question, (2) identifying relevant studies, (3) study selection, (4) charting the data and (5) collating, summarising and reporting the results. These stages form the basis of the methods and results section of this review.

### Research question

The intention of this scoping review is to answer the question, ‘What is the current literature on simulation‐based education for medical radiation students’? For this review, we refined our search strategy based on a PICO approach, where P (population) is the medical radiation student/curriculum, I (intervention) is simulation‐based education, C (comparator) is other forms of learning and O (outcome) is knowledge retention/satisfaction/perceptions/experiences.

### Search strategy

A scoping search was performed on three databases: PubMed, Scopus and Medline from 2010 to 2021. These databases were selected to capture the existing literature in allied health and higher education. To identify the search terms, a preliminary search was conducted in the Scopus and Medline databases. The following terms were entered: ‘simulation’, ‘simulated learning’, ‘computed tomography’, ‘medical radiation’, ‘medical imaging’, ‘radiation therapy’, ‘nuclear medicine’, ‘radiologic technology’ and ‘radiography’. Later, synonyms for each search term were used and applied with the Boolean operators ‘AND’ and ‘OR’ to capture all possible relevant articles (see Table 1). Although no relevant MeSH terms exist for such keywords, these were deemed relevant to the research aims. The search included all peer‐reviewed primary research studies using qualitative and quantitative designs that have been published in English between 2010 and 2021. The timeframe was selected in accordance to the recommendation by Joanna Briggs Institute,^14^ as a narrow timeframe might severely limit the number of eligible studies.

**Table 1:** Databases, search terms and number of hits.

Database	Search terms	Number of hits
PubMed	((radiography[Title]) OR (computed tomography[Title]) OR (medical imaging[Title]) OR (radiation therapy[Title]) OR (nuclear medicine[Title]) OR (radiologic technology[Title]) OR (medical radiation[Title])) AND ((simulation[Title]) OR (simulated learning[Title]))	233
Scopus	TITLE(((radiography) OR (computed tomography) OR (medical imaging) OR (radiation therapy) OR (nuclear medicine) OR (radiologic technology) OR (medical radiation)) AND ((simulation) OR (simulated learning))) PUBYEAR AFT 2010	586
Medline	((radiography) OR (computed tomography) OR (medical imaging) OR (radiation therapy) OR (nuclear medicine) OR (radiologic technology) OR (medical radiation)) AND ((simulation) OR (simulated learning))	232

**Figure 1:**
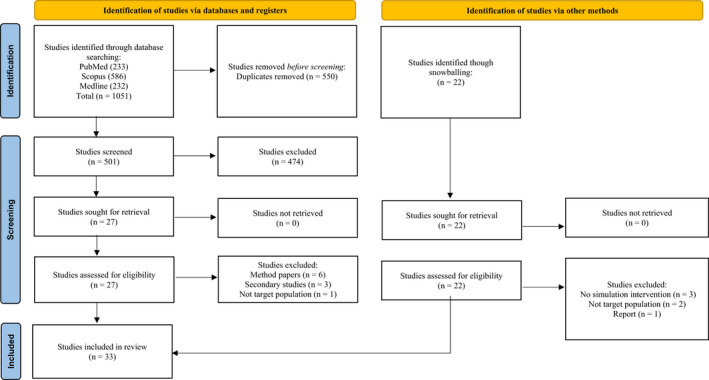
2020 PRISMA flow diagram.

Following the addition of studies identified through snowballing and reference list searching, duplicate studies were removed by a single researcher and titles and abstracts were screened according to the inclusion and exclusion criteria (see Table 2). The independent screening and reviewing of eligible studies was consistent with the 2005 scoping review framework by Arksey and O'Malley,^13^ as well as the Preferred Reporting Items for Systematic Review and Meta‐Analysis (PRISMA) guidelines.^15^ This process has been visually represented using the 2020 PRISMA flow diagram template in Figure 1. Any disagreement was discussed and resolved by consensus among the team members. The research team also had extensive experience conducting scoping reviews, systematic reviews and meta‐analyses, which they used to inform their practice on this reviewing literature.

**Table 2:** Inclusion and exclusion criteria.

Inclusion criteria	Exclusion criteria
Peer‐reviewed papers using simulation education. Reported the use of simulation learning in medical radiations. Published in English between 2010 and 2021.	Only evaluated the software/equipment/instruments. Conference abstracts, case–control studies or case series. Outside the scope of the medical radiation curriculum. Narrative/systematic/scoping reviews or meta‐analysis.

## Results

Table 3 summarises the characteristics of all included studies. Publication dates span from 2010 until the four most recent studies in 2021, highlighting the contemporary nature of simulation. The majority of studies were conducted in developed English‐speaking nations (AUS = 12, UK = 9, IRE = 2, NZ = 2 and USA = 1), with the remaining conducted in the UAE, Finland, Sweden, Norway, France/Switzerland and Portugal. Twelve studies presented quantitative findings, while seven adopted a wholly qualitative approach. Another 14 studies adopted an approach combining both paradigms. Outcomes were most commonly measured based purely from the self‐reported perception of participants (*n* = 30), with Likert scale questionnaires being the most popular tool (*n* = 21). Only seven studies incorporated performance‐based measures to assess skills or knowledge in their data collection. In two of these studies, however, performance‐based assessments were not a prominent feature. Six studies also employed a control group which did not experience the simulation intervention, while one additional study utilised a crossover study approach. None of the studies with a control group employed blinding, though it is noted that effective blinding is largely inconceivable. The total sample size of participants across the studies was 2343, with individual sample sizes ranging from five to 293. ‘Radiography’ was the sole focus for 20 articles, while seven had an interprofessional focus. The remainder focused on a combination of ‘radiation therapy’ (*n* = 5) or ‘sonography’ (*n* = 1). Role play simulation was the most common intervention (*n* = 16) followed by virtual/digital simulation (*n* = 13). Two studies each used simulation video clips or online learning environments as interventions.

**Table 3:** Data extraction table.

Author	Journal	Location	Design	Field	Intervention	Time frame	Outcome(s)	Instrument(s)	Key Finding(s)
Ahlqvist et al. 2013^16^	*Simulation In Healthcare*	SWE	Quant.	MI	Virtual radiography simulator	60 mins (one session/day)	Virtual sim. is effective for teaching image quality assessment.	Knowledge‐based MCQ survey	The interv. group had significantly ↑ scores post‐interv. The control group had no significant change after conventional teaching.
Alinier et al. 2014^9^	*Clinical Simulation In Nursing*	UK	Quant.	MI, RT, physiotherapy, nursing, midwifery, paramedic science, social work, pharmacy	IP sim.	4‐h session (over 3 years)	The interv. enabled students to gain knowledge of professions outside of their own and appreciate IP learning.	Likert Scale questionnaire, knowledge questionnaire	The interv. group reported ↑ perceived knowledge and confidence in working in a team compared to the control group (*P* < 0.05). The interv. group scored 3.23% ↑ than the control group in the discipline knowledge questionnaire (*P* < 0.05).
Bleiker, Knapp & Frampton 2011^23^	*Radiography*	UK	Both	MI	Sim. videos	Unspecified (self‐directed)	The interv. is a useful edu. tool in teaching MI students.	Likert Scale questionnaire, interviews	The interv. gave students insight into patients’ feelings through observation and reflection. Helped students visualise situations they have not experienced yet. Helped relate patient care theory to their clinical practice.
Booth & Kada 2014^38^	*Radiography*	NOR	Quant.	MI	Edu. sessions, role play sim.	2 days of workshops	The interv. can significantly impact the attitudes of student radiographers towards the older population.	Likert scale questionnaire	Attitudes of students were + towards older patients before the interv. Students had a significantly ↑ + attitude towards the elderly after interv. Only a few students (5/38) reported ↑ negative scores post‐interv. (still were overall +).
Bridge et al. 2015^5^	*Radiography*	AUS	Both	RT	VERT	Unspecified	Sim. of the RT workflow and its tasks are feasible using VR sim. applications and software.	Likert scale questionnaire, open‐ended questions	The interv. saved substantial time for academic staff. Students had + feedback regarding its ability to prepare them for clinical placements. A ‘safe’ environment and opportunity to understand clinical workflow before actual clinical experience were important to students.
Brown, Howard & Morse 2016^10^	*Journal of Interprofessional Care*	UK	Quant.	MI, nursing, medicine	IP sim.	Unspecified (over 3 weeks)	The interv. is an effective at preparing students to understand the roles of themselves and others in the trauma team.	Likert scale questionnaire	Students ↑ in preparedness to perform their role and had ↑ understanding of the roles in a trauma setting (*P* < 0.01).
Buckley et al. 2012^11^	*Journal of Interprofessional Care*	UK	Quant.	Medicine, nursing, physiotherapy, MI	IP sim.	Half‐day sessions	The interv. improved understanding of roles and responsibilities, enhancing teamwork and communication.	Likert scale questionnaire, open‐ended questions	Students ↑ in experience, knowledge of other professional roles and the patient’s condition. Students ↑ confidence in interacting with other professional groups.
Carramate et al. 2020^31^	*JMIRS*	POR	Quant.	MI, RT	Role play sim.	One session/day	The interv. enabled acquisition and consolidation of content, highlighting the importance of engaging in their edu.	Likert scale questionnaire	Students valued the learning experience. Students strongly agreed that the interv. aided their learning and helped them acquire, consolidate and deepen their knowledge.
Dungey & Neser 2016^30^	*JMRS*	NZ	Both	RT	Role play sim.	One session/day	The use of high‐fidelity sim. can help develop communication skills and prepare RT students for the clinical environment.	Likert scale questionnaire, open‐ended questions, interviews	Students benefitted from active discussions with peers, actors and staff post‐interv. The realistic sim. scenarios (due to trained actors) helped prepare students for clinical environment. Students demonstrated self‐awareness but not high levels of self‐reflection. Students viewed the interv. as effective, useful and engaging, but were less comfortable receiving feedback from peers. Creating a safe learning environment is important in each sim.
Elshami & Abuzaid 2017^32^	*JMIRS*	UAE	Both	MI	Virtual MRI Simulator	6 × 1‐h sessions	The study supports sim. in MRI edu. and shows that the simulated sessions can be received well by MI students.	Likert scale questionnaire, focus groups	The interv. was effective at providing a comfortable environment for learning. 69% of students used the skills learnt during sim. in their clinical practice. Sim. training aided identification of areas of improvement and helped them learn from mistakes (60%).
Gunn et al. 2018^8^	*Interactive Learning Environments*	AUS	Quant.	MI	Virtual radiography simulator	Unspecified (self‐directed).	VR sim. can enhance the acquisition technical skills of MI students.	Assessor rubric	Mean role play score was significantly ↑ in the interv. group compared to the control (*P* < 0.017). These results translate to a 4.75% improved skill level in favour of the interv.
Gunn et al. 2021^24^	*JMRS*	AUS	Both	MI, RT	Virtual CT simulator	Unspecified	Use of virtual CT sim. does not provide a disadvantage to the students’ confidence.	Likert scale survey, open‐ended questions	93% MI students found the interv. easy to use. 75% MI students enjoyed the interv. The perceived usefulness was + for 57% of the MI students, 36% neutral. 46% were negative/neutral to one of the three categories of usefulness, enjoyment and/or ease of use. 68% of RT students found the inclusion of VR CT sim. helpful. Access to this sim. was beneficial to the student’s clinical CT confidence.
Halkett, Mckay & Shaw 2010^33^	*Radiography*	AUS	Quant.	MI	Edu. sessions, role play sim.	Weekly sessions (3 weeks)	The interv. is an effective method of developing students’ communication and history taking skills.	Likert scale questionnaire	Students were highly satisfied with the workshops. 7/15 items relating to confidence and patient communication had statistically significant increases (*P* < 0.05).
Holmstrom 2019^25^	*JMIRS*	FIN	Qual.	MI	Role play sim. (manikin)	38‐h total	The interv. was a useful learning method in teaching students to perform plain X‐ray examinations.	Observations & interviews	Themes included: ↑ theory‐practice connection, guiding students to follow instructions and strengthening collaboration between students.
Jimenez et al. 2018^21^	*JMRS*	AUS	Both	RT, medical physics	VERT	4‐h (one session/day)	The interv. is an appropriate edu. tool at promoting IP collaboration between RT and MP students.	Likert scale questionnaire, open‐ended questions	Scores showed an insignificant difference in mean scores post‐interv. Satisfaction with VERT was high in both the interv. and control groups.
Lee et al. 2020^17^	*Radiography*	AUS	Both	MI	Virtual CT simulator	1.5 h (self‐directed)	CT knowledge acquisition via remote access with peer‐assisted learning is comparable to local access with facilitation.	MCQ & short‐answer knowledge assessment tests, Likert scale & open‐ended survey	There was no significant difference in CT knowledge between the interv. and control groups. Significant ↑ was seen in assessment scores from the pre‐ and post‐clinical period in both groups.
Leong, Herst & Kane 2018^20^	*JMRS*	NZ	Both	RT	VERT	Two teaching periods	VERT is able to help RT students visualise and connect concepts to the clinical context, giving merit to integrated teaching approaches.	Likert scale questionnaire, open‐ended questions, interviews	Students generally found VERT relatable and beneficial to their learning (93% interested in more VERT sessions). VERT had more benefits to connecting theory to practice through visualisation, standard teaching methods were more valuable for core content. VERT seemed to promote student engagement more than standard teaching. Neither teaching approach was superior, rather they complimented each other.
Liley et al. 2020^22^	*Interactive Learning Environments*	AUS	Both	MI	Virtual CT simulator	2‐h (self‐directed)	CT sim. can not only be engaging but also present challenges, despite still being perceived as inferior to real clinical experience.	Likert scale questionnaire, open‐ended questions	Students had mixed satisfaction. There was a significant ↓ in students’ confidence in their skills after clinical placement. There was ↓ satisfaction from remote learning. There was ↑ preference for hands‐on experiences. Sim. did not help prepare them for clinical practice in terms of CT skills (68% disagreed/strongly disagreed).
Mc Inerney & Baird 2015^35^	*Radiography*	AUS	Both	MI	Online clinical sim. environment	Unspecified	The interv. facilitated acquisition of evidence‐based skills and established reflective practice in students.	Likert scale questionnaire, open‐ended & yes/no survey	57.5% of students found the interv. useful when formulating action plans from clinical situations. 52.55% reported the interv. was effective in bridging theory and practice. The interv. promoted aiming for best practice in a clinical setting. 70% agreed that the interv. ↑ confidence in making professional decisions in clinical situations. 60% felt in charge of their learning.
Naylor, Harcus & Elkington 2015^26^	*Radiography*	UK	Qual.	MI	Role play sim.	One session/day	Use of a service user as a patient in a sim. exercise for student assessment was successful in this setting.	Focus groups	The interv. was valuable for developing and assessing positioning skills, patient care and communication.
Naylor & Foulkes 2018^39^	*Radiography*	UK	Qual.	MI	Role play sim	Two sessions	Sim. is useful for preparing students to work in an operating theatre.	Focus group	Issues in identification and lack of clarity in communication were important in the operating theatre. Lack of preparation of the working environment was also highlighted.
O'Connor et al. 2021^37^	*Radiography*	IRE	Both	MI	Virtual radiography simulator	4 x 30min sessions (self‐directed)	The interv. is a valuable teaching tool in MI edu.	Likert scale questionnaire, open‐ended questions	58% of students enjoyed VR sim. Students ↑ confidence in anatomical marker placement (63%), beam collimation (75%), exposure parameter selection (56%) and centring the X‐ray tube (64%). 55% of students advocated for the use of VR in formative assessments.
Paalimaki‐Paakki et al. 2021^36^	*Radiography*	FIN	Qual.	MI	Virtual coronary CTA sim. environment.	Self‐directed (2 weeks)	The interv. can usefully complement the current counselling practices.	Face‐to‐face & phone interviews	Students felt the interv. provided useful information and familiarisation with the cCTA unit, particularly regarding the department, examination room and scanner.
Reid‐Searl et al. 2014^27^	*JMRS*	AUS	Qual.	MI, sonography	Role play sim.	2 days (40‐min sessions)	The interv. contributed to the clinical communication skills of students.	Focus group	Key themes included: benefits of interacting with someone other than a student, awareness of empathy, engaged problem solving, learning made fun, therapeutic communication skills and purposeful reflection.
Roberts & Goodhand 2018^28^	*Nursing & Health Sciences*	UK	Qual.	MI, nursing, dietetics, occupational therapy, pharmacy, physiotherapy.	IP sim.	45 min (one session/day)	The interv. could be an engaging and useful IP learning activity.	Focus group	Students learnt important ideas central to the IP edu. curricula such as working together as an IP team. This learning is partly from sim. allowing things to go wrong.
Sapkaroski et al. 2018^40^	*JMRS*	AUS	Quant.	MI	Virtual radiography simulator	45 min (one session/day)	Clinical and technical skills is better developed in sim. with dynamic patient interaction than without dynamic interaction.	Likert scale questionnaire	Student perception scores indicated a significant ↑ favouring the VR sim.
Shanahan 2016^7^	*Radiography*	AUS	Quant.	MI	Virtual radiography simulator	Weekly sessions (one semester)	Virtual radiography sim. is valuable in developing technical and cognitive skills.	Likert scale & open‐ended survey	83% found the interv. easy to use. 89% were able to control the equipment as needed. 95% of students benefited from repeating activities until satisfied. 94% benefited from being able to quickly see images and understand if changes needed to be made. 78% reported the sim. developed their technical skills. 85% reported it improving their image evaluation. 85% improved their problem solving. 88% improved self‐evaluation abilities.
Shiner & Howard 2019^41^	*Radiography*	UK	Both	MI	Role play sim.	One session/day	The interv. was effective in preparing students to understand their role within the complex care setting.	Questionnaire with VAS, focus group	Students significantly ↑ in perception of preparedness. Students felt better prepared to perform their role in the imaging of complex care patients.
Shiner 2019^19^	*Radiography*	UK	Both	MI	Role play sim.	One session/day	The interv. provided an opportunity for the students to explore and reflect on initial reactions to new experiences.	Questionnaire with VAS, interview, focus group	The sim. ↓ negative feelings. Emotional preparedness, excitement and distraction ↑. Themes included: building relationships, emotional engagement, developing professional, self‐engagement with wound and sim. impact.
Stowe et al. 2021^18^	*Radiography*	IRE	Quant.	MI	Virtual CT simulator	One session/day	The interv. improved student learning when used as a component in CT edu.	Knowledge‐based multiple choice questionnaire	Mean scores for understanding image quality and dose ↑ after the interv. The interv. proved to be beneficial (although not as significant) when part of a larger CT module.
Titzer, Swenty & Hoehn 2012^29^	*Clinical Simulation In Nursing*	USA	Both	MI, nursing, occupational therapy, respiratory therapy.	IP sim.	One session/day (over 16 weeks)	The interv. provided an environment supporting interdisciplinary teamwork and working in a clinical situation with peers.	Likert scale questionnaire, open‐ended questions	The interv. supported interdisciplinary team work. Independent problem solving was facilitated by allowing students to explore various paths of delivering patient care.
Williams et al. 2015^34^	*Journal of Compassionate Health Care*	AUS	Quant.	Allied Health	Sim. videos	2‐h session	Self‐reported empathy levels can be improved after attending DVD sim. workshops.	Likert scale questionnaire	Mean empathy levels significantly ↑ after interv. MI had the second lowest mean pre‐test empathy score (104) but ↑ post‐interv. by the most (14). No students had a ↓ in empathy scores post‐interv.
Zorn et al. 2019^42^	*Radiography*	FRA/CHE	Qual.	MI	Role play sim.	Three sessions	The interv. had a + impact on the motivation of the students.	Interviews & observations	Sim. sessions were effective in developing high motivational dynamics for students.

+ – positive.

↑ – increase/higher.

↓ – decrease/lower.

AUS, Australia; Edu., education; FIN, Finland; FRA, France; Interv., intervention; IP, interprofessional; IRE, Ireland; JMIRS, Journal of Medical Imaging and Radiation Sciences; JMRS, Journal of Medical Radiation Sciences; MI, medical imaging; NOR, Norway; NZ, New Zealand; POR, Portugal; Qual., qualitative; Quant., quantitative; RT, radiation therapy; Sim., Simulation; SWE, Sweden; CHE, Switzerland; UAE, United Arab Emirates; UK, United Kingdom; USA, United States of America; VAS, Visual Analogue Scales; VERT, Virtual Environment for Radiation Therapy; VR, virtual reality.

The use of performance‐based outcome measures, as adjudicated by external observers or questionnaires was only a major part of the data collection in five studies.^8^, ^9^, ^16^, ^17^, ^18^ Each of these five studies featured a control group which received either conventional educational interventions or no intervention. All studies using performance‐based outcome measures reported significant improvement in favour of simulation other than Lee, Baird,^17^ where no significant difference was found. In this study, the control group received conventional teaching methods, with both groups significantly improving in their core CT knowledge.

Seventeen of the nineteen studies analysing self‐reported quantitative data, demonstrated an increase in competence after completing the simulation intervention. Students reported benefits in areas including empathy, attitudes towards patients, preparedness, confidence, content knowledge, reflection and technical skills. A control group was not utilised in 95% of studies, with Shiner^19^ being the outlier. Leong, Herst^20^ however, employed a crossover study design contrasting conventional teaching methods to VERT, finding that an integrated teaching approach may be of most benefit to the students. Only Jimenez, Thwaites^21^ and Liley, Ryan^22^ identified either no significant difference or decreased perceived competence post‐intervention. Liley, Ryan^22^ reported a significant decrease in the students’ perception of confidence in their clinical skills after the intervention with 68% indicating that simulation did not help them to prepare for their clinical placements.

The studies including qualitative findings used many methods during data collection, namely open‐ended questions (*n* = 10), interviews (*n* = 8), focus groups (*n* = 7), observations (*n* = 1), with five studies employing a combination of methods. Their findings were supportive of the use of simulation, citing enhanced student knowledge, confidence, clinical competence and collaboration with others as positive outcomes. Students mentioned that the opportunity to perform activities relating to positioning, visualisation, communication, clinical preparation, patient care, collaborative learning and relationship‐building were particularly beneficial.^19^, ^20^, ^21^, ^23^, ^24^, ^25^, ^26^, ^27^, ^28^, ^29^, ^30^

The use of simulation as an intervention was received positively by the students in 16 of the 17 studies reporting on satisfaction levels, with only Liley, Ryan^22^ receiving substantial negative feedback. The students in studies by Carramate, Rodrigues,^31^ Elshami and Abuzaid^32^ and Halkett, McKay^33^ agreed that simulation was able to positively impact on their learning and is an important educational tool, endorsing its use into the future.

## Discussion

The review of the literature highlighted key aspects of simulation education, being the influence of type (e.g. roleplay and digital simulation); the capacity of simulation to achieve a variety of outcomes (e.g. clinical skills and preparedness); the mode of delivery (e.g. self‐directed and teacher‐led) and student satisfaction.

All studies included in this review explored simulation as a means for education in a tertiary setting for medical radiation sciences; however, two primary subgroups emerged with regard to the intervention used; role play simulations and virtual/digital simulation. Bleiker, Knapp^23^ and Williams, Brown^34^ both used video clips while Mc Inerney and Baird^35^ and Paalimäki‐Paakki, Virtanen^36^ employed an online learning environment as a means to simulate the clinical setting.

The role play simulation studies can be broken down into further subgroups; practical targeted simulation and interprofessional simulation. For the purpose of this study, ‘practical targeted simulation’ will refer to any simulation‐based teaching approach that was given to a specific population of students, whereas ‘interprofessional simulation’ will refer to any simulation‐based teaching approach given to students as part of a multidisciplinary team. Practical targeted simulation was the intervention of choice for eleven studies, eight of which were specific to radiography participants. The other three studies included participants from radiation therapy^30^, ^31^ and sonography programs.^27^ Six studies simply simulated the clinical environment with the use of role play, three of which incorporated actors to enhance realism.^26^, ^30^, ^33^ Four studies used practical effects such as masks, suits and moulage in order to increase realism in the simulation, with Holmstrom^25^ the only study to use a manikin. These practical targeted simulations proved capable of changing perceived attitudes towards the ageing population and helped to consolidate and deepen knowledge. Further to this, the interventions enhanced student communication, preparedness, clinical skills and promoted self‐reflection. It is noteworthy that the use of actors and practical effects was received well by the students, assisting them to suspend disbelief and fully engage in the activity.^30^ Interprofessional role play accounted for five of the studies, in which participants were involved in a multidisciplinary, situational simulation. This intervention was met with positive feedback from the participants, citing increased levels of confidence, teamwork and better understanding of roles as its benefits. Alinier, Harwood^9^ was the only study to incorporate a control group and measure outcomes based on knowledge gained, finding that the intervention group scored 3.23% higher in the knowledge‐based questionnaire post‐intervention. Students often have their first exposure to interprofessional environments such as trauma or ward radiography during clinical placement and are likely to feel unprepared in the absence of formal training.^10^ Overall, studies which offered interprofessional simulation were seen to be beneficial for preparing students, which could have potential future implications for graduates as they enter the workforce and must work collaboratively with other professions to provide higher quality care.

The intervention that was most common among the virtual simulation studies was virtual radiography software (*n* = 5), allowing the students to position patients and operate an X‐ray tube in a digitally simulated clinical environment. Similarly, four studies used virtual Computed Tomography (CT) software, three used VERT^5^, ^20^, ^21^ while Elshami and Abuzaid^32^ used virtual Magnetic Resonance Imaging (MRI) software. These studies viewed virtual simulation as an effective educational tool. Many noted that it provided the students with a safe environment to make mistakes and learn while also preparing the students for their clinical placements. Leong, Herst^20^ reported increased engagement when contrasted to conventional teaching methods; however, they did not identify any significant benefits to achieving learning outcomes. Rather, its real benefit lies in integrating the two learning models. Student satisfaction remained positive throughout these studies with common responses indicating that the experience was beneficial to their education. Self‐reported improvement was seen in many categories including understanding of image quality, dose, critical thinking, image evaluation and clinical skills. Students enjoyed having free access to the software to work at their own pace with less stress while developing familiarity in a clinical context. Having a safe environment to repeat examinations and learn from their mistakes were also positive outcomes. Conversely, confusing software, technical difficulties and lack of support led to some negative experiences. One study by Liley, Ryan^22^ noted mixed results among the students with a decrease in their perceived clinical skill levels. The participants expressed a desire for ‘hands‐on’ experience in preference to remote access learning.

Simulation video clips were found in one study to significantly increase empathy levels in an interprofessional context.^34^ Although radiography students exhibited the second lowest empathy levels in the pre‐test measurement, medical radiation students (radiography and radiation therapy) benefitted the most from the intervention. Similarly, Bleiker, Knapp^23^ also noted themes of increased empathy as well as linking theory to practice, demonstrating that simulation videos can be an effective tool in medical radiation.

Although quite different in execution, both studies involving online learning environment simulations allowed the students to experience the clinical environment and learn remotely. Mc Inerney and Baird^35^ demonstrated that most students (70%) believed the simulation to be beneficial to their professional judgement and clinical decision making; however, only 52.55% reported that the simulation was an effective link between theory and practice. The students participating in the study by Paalimäki‐Paakki, Virtanen^36^ found the interactive environment was suitable for familiarisation of the department and equipment in the clinical context, but did not explore this in great detail. As only two studies were found utilising this intervention, it makes it difficult to draw conclusions. Further studies with similar methodologies and interventions are warranted.

Student outcomes across all studies were generally positive towards simulation. The studies using performance‐based outcome measures demonstrate its capability to achieve a variety of outcomes ranging from theoretical knowledge to clinical skills. Each of these studies reported statistical significance in the improvements over the control group, highlighting the advantages of simulation over conventional teaching methods. The favourable results from Alinier, Harwood^9^ and Stowe, O'Halloran^18^ reflect well on their respective interventions; however, their control groups received no intervention. This fails to address the question regarding the effectiveness of simulation compared with conventional teaching methods.

Similarly, the self‐reported benefits from the students demonstrate the versatility of simulation to achieve a desired outcome. While only a few studies employed control groups, the results show that most students are able to reflect on the intervention and identify benefits to their learning. Although this is less rigorous than other methodologies, outcomes such as preparedness and confidence are difficult to assess via alternate means without participant bias. Of the two studies receiving mixed qualitative responses, both used CT virtual simulation as the intervention. These responses were primarily due to the unfamiliar systems and lack of support but were also influenced by the lack of interaction with a physical CT environment.^17^, ^22^ It is important to note that although the benefit of simulation is clear, most studies are of the opinion that it should complement clinical placement rather than replace it.^5^, ^22^, ^27^, ^37^ This is in accordance with Thoirs, Giles^6^ where it was the view of tertiary educators, accrediting bodies and clinicians that simulation should not replace clinical placement.

Students commonly reported that they enjoyed the simulation and that similar experiences should be incorporated into their respective courses. A large factor for this was the capacity for self‐directed learning for online simulations whereby the students could complete the tasks in their own time. The high‐fidelity nature of many simulations was also a contributor to the satisfaction levels.^26^, ^30^, ^38^ The lack of control groups in these studies may again skew the results in favour of the intervention as the students had no comparative teaching method. Liley, Ryan^22^ was the only study to report mixed satisfaction levels within the students. This was primarily due to the remote‐access nature of the intervention leading to frustration within the participants and was also seen to a lesser extent in other virtual interventions.^37^ However, it is important to note that this was a pilot study with a relatively small sample size.

### Limitations

The studies comprising this review primarily relied upon self‐reported outcome measures which are considered much less reliable than objective measures. Quantitatively determining the effect of simulation interventions should be prioritised by employing objective outcome measures in future research. Control arms should also be included in future research where possible to improve methodological quality. It should be noted that many institutions would employ simulation but may not publish their practices. Additionally, publication bias may have impacted the results as there was no active search of grey literature (e.g. unpublished theses and conference proceedings), and this review only included English‐language studies. Publication of studies with more favourable results are more likely to be published than those with contrary findings, meaning that the literature available may overestimate the true value of simulation interventions. Real‐world outcomes such as cost were not reported in any included study. Data regarding costs of implementation and qualitative discussion concerning accessibility of resources would be advantageous in enabling financial and resource analysis of given interventions.

## Conclusion

It is evident that the use of simulation‐based education can have significant effects on the learning of students in medical radiation. Almost all studies included in this review viewed the use of simulation in Medical Radiation education positively. If implemented appropriately, simulation can provide students with opportunities to experience the clinical environment in a safe context and learn at their own pace. Both practical and virtual simulation have shown their potential in a variety of contexts in this review, with many students endorsing its use in medical radiation courses as a complementary learning tool rather than a replacement for clinical practice. Due to the small number of studies with objective performance‐based outcome measures and control arms, it is difficult to arrive at a reliable comparative evaluation of the relative benefits of simulation versus traditional teaching methods. Nevertheless, this review highlights the benefits of simulation in medical radiation education and outlines the shortcomings in recent literature. There is a need for further research into simulation using objective outcome measures and control arms, particularly concerning modalities such as CT and MRI.

## Conflict of interest

The authors declare no conflict of interest.
